# Congestion of the Kidneys

**Published:** 1841-01

**Authors:** 


*Congestion of the Kidneys.*—Dr. Watson reported the case of a sailor who entered the hospital for retention of urine without stricture. On passing the catheter, no urine was found in the bladder; high febrile excitement ensued, the bowels became tympantic, and the patient died two days after admission. On autopsy, both kidneys were found enlarged, and studded in their external surface with numerous spots of coagula. Dr. W. had never met with a similar case.—*N. Y. Jour. of Med. and Surg. Ib.*

